# #StayHome: Monitoring and benchmarking social isolation trends in Caruaru and the Região Metropolitana do Recife during the COVID-19 pandemic

**DOI:** 10.1590/0037-8682-0271-2020

**Published:** 2020-06-24

**Authors:** Patricia Takako Endo, Ivanovitch Silva, Luciana Lima, Leonardo Bezerra, Rafael Gomes, Marcel Ribeiro-Dantas, Gisliany Alves, Kayo Henrique de Carvalho Monteiro, Theo Lynn, Vanderson de Souza Sampaio

**Affiliations:** 1 Universidade de Pernambuco, Recife, PE, Brasil.; 2 Universidade Federal do Rio Grande do Norte, Natal, RN, Brasil.; 3 Institut Curie, France.; 4 Dublin City University, Ireland.; 5 Fundação de Medicina Tropical Doutor Heitor Vieira Dourado, Manaus, AM, Brasil.; 6 Fundação de Vigilância em Saúde do Amazonas, Manaus, AM, Brasil.

**Keywords:** COVID-19, Social isolation, Public health, Public health policy

## Abstract

This technical report presents information related to the Social Isolation Index (SII) of the city of Caruaru, Pernambuco, Brazil. The data was provided by In Loco, a technology startup that has collected the movement of around 60 million Brazilians through cell phone location.

## INTRODUCTION

COVID-19 is a pandemic characterized by uncertainty in transmission, pathogenicity, and strain-specific control options. Governments have a wide range of non-pharmaceutical public health intervention strategies to mitigate the transmission and the associated socio-economic effects of respiratory diseases, including hand-washing, use of face masks, closure of educational institutions and non-essential businesses, transport and travel restrictions, and the focus of this report, i.e., quarantine, self-isolation, and social distancing^1^. The success of such strategies depends on the effectiveness of government authorities in strategizing communication and enabling measures and both personal and community voluntary responses and behaviors^2^. Mathematical models are widely used for informing government decisions. However, lessons from previous coronavirus outbreaks, for example severe acute respiratory syndrome, is that the proportion of transmission that occurs before the onset of clinical symptoms or in asymptomatic infection could be as important as the intrinsic transmissibility for determining the ease of control and effectiveness of intervention strategies^3^. Data on the impact of government interventions are difficult to collect and if collected at all, may not be available to local decision-makers at the level of granularity to make timely decisions and interventions during a rapidly evolving pandemic. Consequently, a simple, rapid, low-cost, time- and context sensitive indicator of the effectiveness of intervention strategies, such as social distancing is required. In the absence of mobility data from official sources, anonymous mobile phone traces can be used as a proxy of human movement and social interactions^4^ ^,^ ^5^. 

This technical report uses data from In Loco, a Brazilian consumer geo-tracking and advertising software vendor, to create a proxy for effective social distancing rates based on consumers’ spatio-temporal movements, and then uses these measurements to create a social isolation index (SII)^6^. Furthermore, the granularity of data available may allow targeted interventions in smaller, well-defined communities or streets, i.e., at a hyperlocal level. To illustrate this, we compared the city of Caruaru in Pernambuco, Brazil with 15 municipalities in the Região Metropolitana do Recife (RMR). 

Caruaru is the most populous city in the interior of Pernambuco with 361,118 inhabitants; it is the most important city in the Agreste Pernambuco^7^ ^,^ ^8^. It is an economic center for textiles and handicrafts, hosting one of the largest handicraft markets in Brazil, the Feira de Caruaru, with over 28,300 vendors in a space over 40,000 m^2^ in size^9^. 

## METHODS

In Loco’s software is installed in over 60 million mobile phones through its integration on approximately 600 partner applications^6^. These applications were downloaded voluntarily by end users under the In Loco or partner terms and conditions, which allow the collection of spatio-temporal data necessary for the functioning of the app and In Loco advertising. Anonymous consumer spatio-temporal data collected by In Loco were used to construct the SII based on the proportion of mobile phones in a given geographic area that remained within a radius of 450 m of their “habitual home” location during a given day. In line with recommendations from In Loco^6^, habitual home is defined as the location with the largest activity at night time, in this case, where the mobile phone is located overnight. A polygon-based approach was used to create geographic boundaries for cities, states, and other geographic objects. Individual-level data were aggregated for pre-defined geographic areas, where a minimum number of In Loco users were active. Once aggregated, the data were visualized using Tableau. There was an inverse relationship between the SII score and commuter movement, i.e., a higher SII score implied less movement from the habitual home and vice-versa. For example, if the SII score is 30% for a specific geographic area on a given day, it indicates that 30% of monitored mobile phones remained within 450 meters of their habitual homes on that day. State-level data are available publicly on In Loco’s website^10^; more granular data are only available subject to agreement with In Loco.

Despite the federal government’s reluctance, and in the absence of readily available alternatives, many states and municipalities are using In Loco data to inform decision-making, including Alagoas, Amapá, Amazonas, Ceará, Maranhão, Goiás, Mato Grosso do Sul, Mato Grosso, Minas Gerais, Pará, Paraíba, Piauí, Santa Catarina, and Rio Grande do Sul^11^. In Loco data are also being used in Recife and Caruaru to monitor social isolation. 

## RESULTS AND DISCUSSION

On March 15, 2020, state public health measures to mitigate the spread of COVID-19 were implemented in Caruaru, including self-isolation, social distancing, quarantine, suspension of education, and gatherings of over one hundred people^12^. The market and all non-essential businesses were closed by March 20, 2020, and mobile health and hygiene controls were installed on March 22, 2020^13^. The first official case of COVID-19 in Caruaru was registered on March 23, 2020, the first case of community contamination on March 27, 2020, and the first death by COVID-19 on April 10, 2020. By May 1, 2020, Caruaru had 59 confirmed cases and five deaths from COVID-19, as well as 131 suggested cases of the disease^13^. 

**FIGURE 1:**
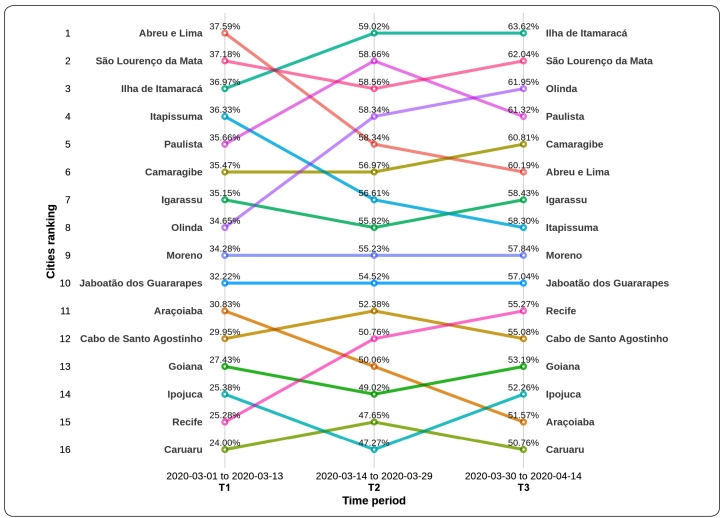
In Loco Social Isolation Index for 15 Região Metropolitana do Recife municipalities and Caruaru (Data Source: In Loco).

Using SII data sourced from In Loco, Figure 1 presents the SII for Caruaru and 15 municipalities in RMR at three points from March 1, 2020 to April 14, 2020, and ranks each by SII based on the hypothesis that higher index scores correlate with higher effective social isolation. T2 corresponds to the day following the order implementing state measures. As can be seen, the SII increased dramatically in all municipalities as state public health measures were implemented, moving from an average SII of 32.4% at T1 to 57.5% at T3; the SII range at T1 was 24%-37.6% and 50.8%-63.6 at T2. 

The SII suggests that movement declined by T2 as general knowledge and media coverage of COVID-19 increased and then decreased again following the implementation of state measures. This suggests that both media coverage and state intervention play important but different roles in supporting social distancing and isolation measures. However, the data also suggest that community response to public health media coverage and interventions is not uniform. Indeed, despite the measures taken locally in Caruaru, even before the first reported case of COVID-19, their SII remained the lowest of the 16 municipalities ranked. This suggests that to ensure effective social distancing and achieve social isolation, ongoing local public health interventions are required. 

## FINAL CONSIDERATIONS

Federal, state, and local public health policy makers and influencers need to measure, monitor, and respond to factors impacting the social and economic life of urban communities. With novel epidemics, uncertainty in the transmission, pathogenicity, and availability and effectiveness of strain-specific control options complicate both decision-making and the efficacy of intervention. Community norms and behaviors are not homogenous, neither are public interventions. Local proactive decision-making and anticipatory interventions require local intelligence so that remediation and optimization actions can be taken. This technical report suggests that spatio-temporal data from existing consumer software applications, although not designed for public health purposes, can be useful for monitoring adherence to public health policies in pandemics. We indicated how such data can be used for monitoring social isolation. 

This is not however a panacea for social isolation monitoring. The sample used for the SII described in this report is not necessarily representative of the general population. At-risk cohorts, including older people and those living in extreme poverty, are less likely to have a mobile phone or have the In Loco software installed. Notwithstanding this, SII is a promising, potentially useful, and necessary indicator for timely and effective social isolation decision-making. Benchmarking allows government officials to identify not only trends but also relative performance and learn from others who would appear to be performing more effectively before taking further action. Policymakers need to consider whether mandating access to similar spatio-temporal data from commercial providers overrides the right to privacy when in the public interest. In future work, we will report on the impact of additional public health interventions and explore the relationship of SII with other demographic variables, media coverage, and COVID-19 statistics for each urban community.
